# Titer on Chip: New Analytical Tool for Influenza Vaccine Potency Determination

**DOI:** 10.1371/journal.pone.0109616

**Published:** 2014-10-20

**Authors:** Laura R. Kuck, Michelle Sorensen, Erin Matthews, Indresh Srivastava, Manon M. J. Cox, Kathy L. Rowlen

**Affiliations:** 1 InDevR Inc., Boulder, Colorado, United States of America; 2 Protein Sciences Corporation, Meriden, Connecticut, United States of America; The University of Adelaide, Australia

## Abstract

Titer on Chip (Flu-ToC) is a new technique for quantification of influenza hemagglutinin (HA) concentration. In order to evaluate the potential of this new technique, a comparison of Flu-ToC to more conventional methods was conducted using recombinant HA produced in a baculovirus expression system as a test case. Samples from current vaccine strains were collected from four different steps in the manufacturing process. A total of 19 samples were analysed by Flu-ToC (blinded), single radial immunodiffusion (SRID), an enzyme-linked immunosorbent assay (ELISA), and the purity adjusted bicinchoninic acid assay (paBCA). The results indicated reasonable linear correlation between Flu-ToC and SRID, ELISA, and paBCA, with regression slopes of log-log plots being 0.91, 1.03, and 0.91, respectively. The average ratio for HA content measured by Flu-ToC relative to SRID, ELISA, and paBCA was 83%, 147%, and 81%, respectively; indicating nearly equivalent potency determination for Flu-ToC relative to SRID and paBCA. These results, combined with demonstrated multiplexed analysis of all components within a quadrivalent formulation and robust response to HA strains over a wide time period, support the conclusion that Flu-ToC can be used as a reliable and time-saving alternative potency assay for influenza vaccines.

## Introduction

Exciting advances in flu vaccine production technology have been realized during the past few years. In 2012, Novartis’ Flucelvax was approved by the FDA as the first flu vaccine produced in cell culture [1] and in 2013, Protein Sciences Corporation’s Flublok was the first recombinant antigen flu vaccine approved by the FDA [2]. Virus-like particles (VLPs) are also being developed as novel flu vaccines with production methods ranging from recombinant antigens produced in insect cell culture (e.g., Novavax) to plant-based platforms (e.g., Fraunhofer and Medicago Inc.). There are also promising advances in the development and production of a “universal flu vaccine” [3].

Despite these innovations in production methods new flu vaccines are still being characterized by conventional analytical methods, as recently noted by Thompson et al. [4]. For example, the single radial immunodiffusion assay (SRID) has been used to quantify flu vaccine potency since 1978 [5] and it remains the FDA- and WHO-approved gold standard method. The SRID assay is an antigen-antibody based assay that relies on seasonal antigens and antisera generated and distributed by Reference Laboratories around the world (e.g., CBER in the US, NIBSC in the UK, TGA in Australia, NIID in Japan). The time required for generation and distribution of reference antisera is recognized as a rate limiting step in influenza vaccine development and characterization [6], [7]. Often, reference antigens can be produced with reasonable expediency but vaccine producers experience significant delays in characterization of their material due to the complexities associated with generating and validating reference antisera. Furthermore, the SRID assay is expensive because it is a time- and labor-intensive assay that takes 2–3 days to complete with a minimum of 6 hours hands-on time by well-trained analysts. While reference reagents are provided by CBER and other Reference Laboratories, gels and other reagents must be prepared in-house and the methodology independently validated by each vaccine producer.

There is an urgent need to “improve or replace the SRID assay [to] facilitate seasonal and pandemic influenza preparedness” [6]–[8]. Alternatives to SRID for quantification of hemagglutinin (HA) protein under non-biologically relevant conditions include HPLC [9], [10] and mass spectrometry [11], [12]. HPLC is widely used in the vaccine industry but has not been adopted as an alternative to SRID due to the non-biologically relevant conditions associated with protein digestion. Mass spectrometry methods provide excellent sensitivity and specificity but also rely on protein digestion and tend to be considered too expensive and too complex for robust use in regulated quality-controlled environments. Potential alternatives to SRID that measure biologically relevant forms of hemagglutinin include surface plasmon resonance detection [13], which offers improved sensitivity relative to SRID but has high capital equipment costs [6], and ELISA-based methods.

Vaccine producers generally rely on an Enzyme-Linked Immunosorbent Assays (ELISAs) as an alternative to SRID [14] for analysis of “in-process” (i.e., crude) samples. ELISA is more rapid than SRID (hours versus days) and meets the requirements of subtype specificity and stability indication [6]. However, in general the method still relies on reference antisera from CBER or costly development of new antibodies each year unless one is able to use universal antibodies. In addition, each lab must produce its own batch of plates, introducing inter-laboratory error [15], [16].

To overcome the limitation of reliance on reference antisera, several groups are working on production and characterization of “universal” antibodies that can be used as capture agents for immunoassays. For example Hashem et al. [17] recently developed a novel sialic-acid based synthetic receptor for use in an immunoassay for influenza hemagglutinin (HA). Li and co-workers [18] developed an antibody that exhibits nearly universal binding to all HAs. Bodle et al. [19] demonstrated that immunoassays with cross-reactive monoclonal antibodies (mAbs) could be used for a number of seasonal variations for each subtype. Similarly, influenza Titer on Chip (Flu-ToC) is a new, multiplexed immunoassay for HA quantification that utilizes a *panel* of subtype-specific but broadly reactive monoclonal antibodies. Multiple antibodies against seasonal A/H1, A/H3, B/Yamagata-like and B/Victoria like strains are printed in an array format. Signal readout is based on fluorescence from a fluor-conjugated “universal” primary antibody label. In this work, a blinded study was conducted to evaluate the potential of Flu-ToC as a robust alternative to SRID for a variety of sample types, from crude extract to bulk drug substance (BDS).

## Materials and Methods

### Sample Collection and Handling

Briefly, the rHA protein was solubilized from the insect cell membrane using Triton X-100 surfactant and released into a buffer yielding crude extract. Cell debris and suspended solids were removed from the cell extract by filtration. The rHA in the filtrate was purified by two chromatography steps and an additional filtration step, and then formulated into phosphate buffered saline (PBS). The proteins were diluted, if necessary, to a final total protein concentration of 500–700 µg/mL based on total protein by the BCA assay. The purified rHA proteins were filtered through a 0.2 µm filter and designated bulk drug substance (BDS).

Samples in the study included aliquots from various steps in the rHA purification process. Crude extract (CE), filtered crude extract (FCE), and Column 1 eluate samples were stored at −20°C. Column 2 eluate and bulk drug substance (BDS) samples were stored at 2–8°C.

### Flu-Toc Antibody Panel

**Figure 1:**
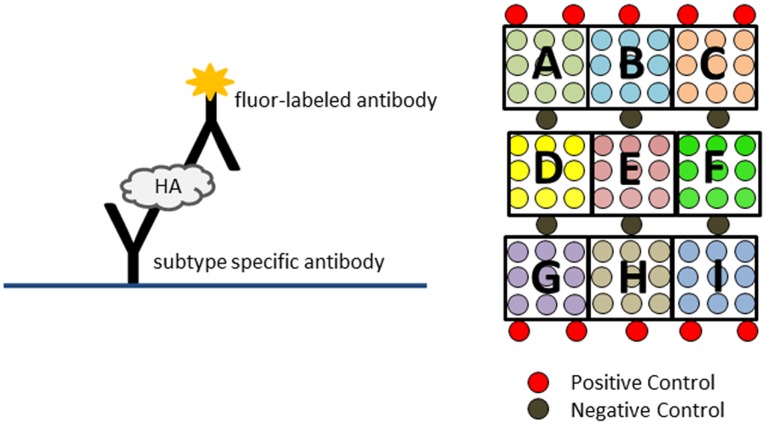
The immunoassay is illustrated in the left panel and the array layout is outlined in the right panel. The array contains 9 replicate spots (∼200 µm in diameter) of each monoclonal antibody (designated A–I). Details for each antibody are given in Table 1.

The assay and array layout for Flu-ToC are illustrated in **Figure 1** **.** HA proteins are captured by sub-type specific monoclonal “capture” antibodies and detected by a universal antibody (polyclonal or monoclonal) conjugated with a “Cy3” equivalent fluorophore (excitation at 532 nm and emission at 570 nm). The array contains 9 replicate spots of each capture antibody. In the current version of the array (v1.0 #5521, InDevR), there are 3 distinct antibodies for A/H1 and A/H3, 2 antibodies for B/Yamagata-like strains, and a single antibody for B/Victoria-like strains. [Note: consistent with the Bayh-Dole act (94 Stat. 3015, 35 U.S.C. 200–212, 37 C.F.R. 401), the antibodies used as reagents in the Titer on Chip product are held as proprietary]. As summarized in **Table 1**, for seasonal A/H1 and A/H3, the array contains a combination of capture antibodies that are conformational and neutralizing mAbs against HA’s globular head (HA1) as well as the highly conserved region of the stem (HA2). In addition, at least one antibody against the linear epitope is included for H1, H3, and B/Yamagata-like subtypes/strains in order to provide additional and complementary data into epitope exposure. For the work described here, only the capture antibodies that are both conformational and neutralizing were used for quantification; specifically, antibodies in positions A, D, G, and I (defined in Figure 1) were used to quantify H1, H3, B/Yam-like, and B/Vic-like subtypes/strains, respectively.

**Table 1:** Antibody Description for Flu-ToC v1.0.

Array Position	Epitope Type	Epitope Location	Neutralizing	Detection Target
A	Conformational	HA1	YES	A/CA/2009 (pdm-like H1)
B	Conformational	HA2	YES	Broad H1
C	Linear	HA1		Broad H1
D	Conformational	HA1	YES	Broad H3
E	Conformational	HA2		Broad H3
F	Linear	HA1		Broad H3
G	Conformational	HA1	YES	Broad B/Yam
H	Linear	HA1		Broad B/Yam
I	Conformational	HA1	YES	Broad B/Vic

The label antibody mixture includes a “universal” polyclonal (#5514, InDevR), which can be used to quantify all components of a quadrivalent HA mixture from split viruses and virus-like particles. However, while recombinant B HA proteins can be detected with the polyclonal label antibody, recombinant H1 and H3 require subtype-specific monoclonal label antibodies. For the detection of H1 HA, an H1-specific monoclonal neutralizing antibody directed against a conformational epitope on the F sub-domain of HA2 was used. For H3 HA, a non-neutralizing monoclonal antibody (#5513, InDevR) that binds to a conserved region of HA2 was used for detection.

### Flu-ToC Method

Version 1.0 of the Flu-ToC kit contains 2 glass slides (3 in × 5 in), each printed with 16 arrays per slide. Typically, 8 arrays on one slide were used for an 8 point calibration with standards and the remaining 24 arrays were used for samples. The array-containing slides were allowed to come to room temperature prior to pre-processing in Wash Buffer 1 (#5532, InDevR) for 5 minutes on an orbital shaker (50 rpm). The slides were ready for use after drying. Since the quantification range is typically below 3 µg/mL, samples were diluted into Blocking Buffer (#5531, InDevR) to yield final concentrations within the quantification range. Sample and standard volumes of 50 µL each were placed directly onto respective arrays and the slide incubated in a humidity chamber at room temperature for one hour. After incubation, sample solutions were removed by pipette and the slides washed in Wash Buffer 1 for 1 minute and Wash Buffer 2 (#5533, InDevR) for 5 minutes. After removal of excess wash solution, the appropriate fluor-conjugated label antibody and positive control label (#5512–15, InDevR) were added and the slides incubated at room temperature for 30 minutes. Excess label solution was removed and the slide was washed sequentially with Wash Buffers 1 and 2. Excess liquid was removed prior to imaging on a fluorescence microarray scanner (Vidia, InDevR). Imaging was conducted using excitation centered at 530 nm and emission at 570 nm with a typical collection time of 1 s per array. With this protocol, 8 standards and 24 samples can be processed within 3 hours.

Calibration was conducted with glycerol reference standards of recombinant HA (Protein Sciences Corporation) from A/CA/07/2009 (H1), A/TX/50/2012 (H3), B/Brisbane/60/2008, and B/Mass/02/2012. These reference antigens had been previously calibrated by SRID against CBER standards. For the blinded study, each sample batch was calibrated against recombinant reference antigens that had been serially diluted into Blocking Buffer to yield an 8 point standard range, including a blank. A typical calibration concentration range was zero to 1–3 µg/mL, with the lowest concentration being ∼0.02 µg/mL.

### Flu-ToC Automated Quantification Algorithm

Quantitative data were extracted from fluorescence images using a custom software package and results were obtained using an automated algorithm. The image analysis program automatically: i) extracts the median digital signal from each spot in the image, ii) determines the median value from each set of 9 replicate spots per capture antibody and 10 replicate positive control spots, iii) determines the median background value from 20 spots, iv) normalizes the analyte signal (median value) to the internal positive control, v) automatically plots and analyzes calibration data from the 8 calibration arrays (fluorescence signal as a function of HA concentration), vi) determines linear response ranges within the calibration curve via linear regression analysis, and vii) uses the linear regression fit to back-calculate HA concentrations for samples.

Normalization of the signal on each array using an internal control was used to account for array-to-array variability during manufacture and (or) processing. The internal control was a mouse monoclonal IgG that is subsequently labeled with a fluor-tagged goat anti-mouse IgG during the labeling step. Specifically, the median background value was subtracted from the median sample value, the result multiplied by 100 and divided by the background-corrected median internal control value. The background value on each array was determined from the median of 20 non-spotted areas at specific locations on each array. The non-spotted areas used for background determination are distinct from the negative controls, which are used as a substrate check. We note that median values are used for all calculations, rather than the mean, as a more effective way to eliminate statistical outliers. Since array processing can sometimes lead to non-relevant “bright spots”, outliers from multiple measurements (9 measurements in the case of samples, 10 for internal controls, and 20 for the background) can be discarded.

The “4 point” linear region approach was used in in order to accommodate typical binding curves without relying on a multi-parameter non-linear regression. Thus, for each calibration curve there may be multiple linear regions. The quantification algorithm automatically assesses four separate criteria to determine the suitability of a particular linear range for a specific sample: specifically, 1) a linear region is defined as 4 adjacent points that yield a Pearson’s correlation coefficient (R) >0.95, 2) a slope >10, 3) the normalized sample signal must fall within the 4 point range, and 4) the normalized sample signal must be above the quantification limit. The quantification limit is defined by the normalized signal for the blank plus a multiple of the standard deviation in the blank, typically 10x. If a sample signal meets the criteria for more than one linear region on a curve, the average value is reported along with the measured precision.

### SRID

Samples and reference standards at 30 µg/mL were incubated in 1% Zwittergent 3–14 for 30 minutes, diluted to final concentrations of 30, 20, 15, and 7.5 µg/mL, and loaded onto agarose gels containing an appropriate polyclonal antibody. Gels were incubated for 18–24 hours, rinsed with 0.9% saline, pressed under approximately 2 kg for 30 minutes to remove water, dried, and then stained with Coomassie brilliant blue R (B0149, Sigma). The precipitin rings were measured using ImmuLab SRID reading instrument and the potency of the sample relative to the standard was determined using parallel line analysis.

### Protein by Purity-Adjusted BCA

Total protein was determined by the Bicinchoninic Acid (BCA) Assay. Bovine serum albumin (23209, Pierce) standards were prepared in concentrations from 1500–25 µg/mL. The standards and samples were loaded onto 96-well plates (15041, Thermo Scientific) in triplicate, then treated with BCA assay reagents (23228 and 23224, Pierce) per manufacturer instructions. After incubating for 30 minutes at 32°C, the plate was read at 562 nm using a BioTek Synergy H5 plate reader and analyzed using BioTek Gen5 software. The standard curve was calculated using a 4 parameter non-linear fit. The sample concentration was determined using linear regression. Total protein results were corrected for purity (designated purity-adjusted BCA, paBCA). Purity was determined by SDS-PAGE densitometry; the ratio of the rHA band to the total of all non-rHA bands was calculated.

### ELISA

Nunc Immuno MaxiSorb 96-well plates (442404, Thermo Scientific) were coated with a sheep-derived polyclonal HA antibody diluted in PBS and then blocked with 4% BSA (A8022, Sigma) in PBS (20012–050, Gibco). After washing the plates in 0.05% Tween 20 (0777, Amresco) in PBS, the plates were loaded with samples and reference standards diluted in PSC formulation buffer to 6 concentrations covering the linear range of the ELISA curve. Each sample and reference was prepared in duplicate and each preparation was loaded onto duplicate plates. The plates were incubated for 1 hour at room temperature while orbiting at 100 rpm. Plates were washed with 0.05% Tween 20 in PBS and then incubated with a polyclonal rHA antibody raised in rabbit in a diluent containing PBS with 0.1% BSA and 0.01% Tween 20. The plates were incubated for 1 hour at room temperature orbiting at 100 rpm. Plates were washed with 0.05% Tween 20 in PBS, and then incubated with α-rabbit IgG conjugated with horse-radish peroxidase (A4914, Sigma). The plates were incubated for 1 hour at room temperature orbiting at 100 rpm. After a final wash, the plates were developed with TMB 1-Component Peroxidase Substrate (53-00-02, KPL) and the reaction was stopped with 1N hydrochloric acid. Plates were read at 450 nm on a BioTek Synergy H5 plate reader and data were analyzed using BioTek Gen5 software. The sample concentrations for each dilution were determined by linear regression using the standard curve.

## Results and Discussion

### Flu-ToC Qualitative Response

**Figure 2:**
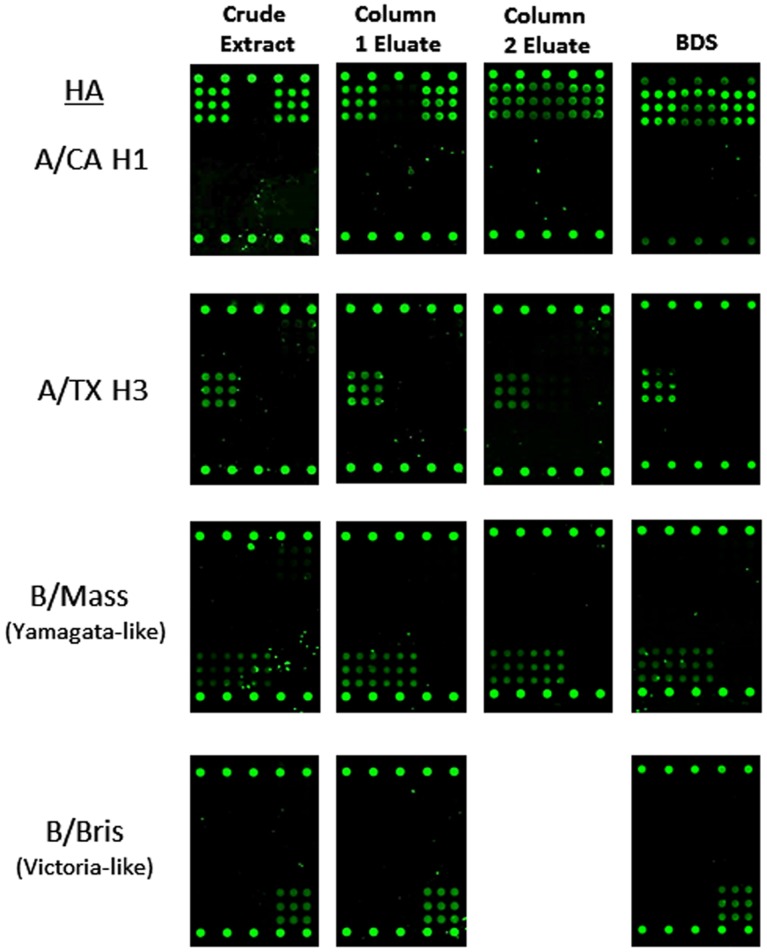
Representative fluorescence images for each rHA (rows) tested in the blinded study. Each step in the manufacturing process is represented in the 4 columns. There was no column 2 eluate sample for B/Bris. For these particular arrays there is detectable cross-reactivity between the pAb label and capture antibody “C”.

One of the primary objectives of this study was to determine whether Flu-ToC could be used for the quantitation of HA at all stages of the manufacturing process, including crude extracts from cell culture where the antigen concentration is low and “contaminant” levels are high. **Figure 2** contains a compilation of representative fluorescence images from rHA antigens from each of the subtypes and different B lineages at each stage in the manufacturing process. The specificity is high for each subtype, including distinct response to B/Yamagata-like and B/Victoria-like, as evidenced by the lack of signal on capture mAbs for other subtypes. Overall, the level of specificity enables analysis of samples in either monovalent formulations or multivalent formulations (multiplexed). As can be observed in the Figure 2 image set, for all rHA antigens Flu-ToC exhibited good signal and minimal background for the crude extracts as well the other process steps through bulk drug substance (BDS). Although not part of this study, we note that Flu-ToC also exhibits good specificity for HA derived from whole virus produced in eggs, with minimal background from allantoic fluid.

### Flu-ToC Quantitative Response

**Figure 3:**
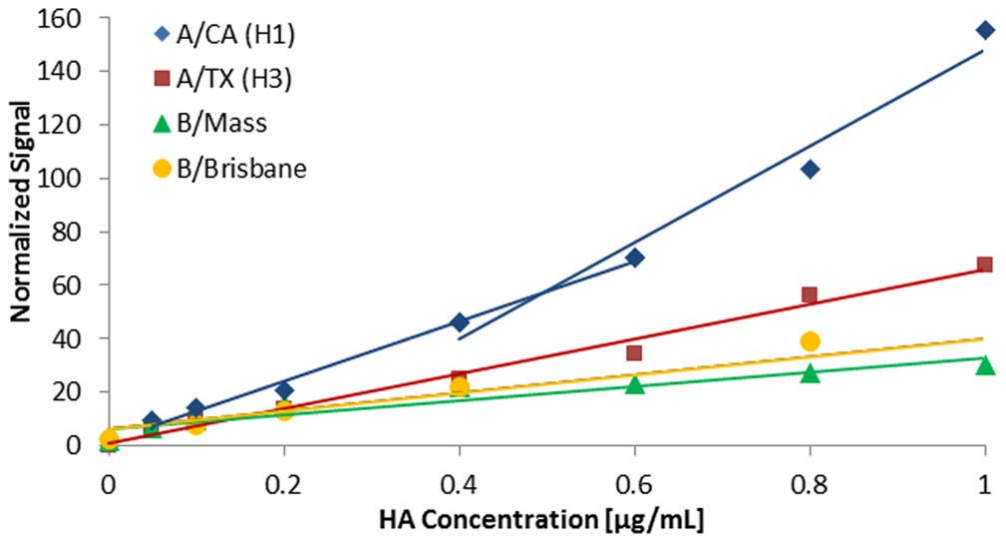
Representative calibration curves for each reference strain (rHA). Two linear 4 point regions for A/CA are included to illustrate the approach employed within the automated algorithm (see text for details).

Representative calibration curves are shown in **Figure 3**. The quantification limits and ranges were determined to be ∼0.025–1 µg/mL for H1 and H3 rHA antigen and ∼0.1–3 µg/mL for B rHA antigen. The sensitivity and range for H1 and H3 are particularly good, with a span of 40x. For comparison, the typical quantification range for SRID is ∼5–36 µg/mL (7x span) and ELISA has a 25x span with a quantification range of 0.004–0.1 µg/mL for H1 California and B/Brisbane and 0.04–1.0 µg/mL for H3 Texas and B/Massachusetts.

**Table 2** summarizes the Flu-ToC results reported to PSC prior to un-blinding of the sample concentrations. The content of rHA antigen for each strain measured at each stage in the manufacturing process is shown in the “ToC” column along with measured error. The values are weighted averages from replicate testing. The average relative error (precision) over all Flu-ToC measurement was 21%; however, not surprisingly, the average relative error was highest for the crude extracts (36%). Excluding crude extracts, the average relative error was 16%. The cleanest samples (BDS) exhibited an average relative error was 12%. ELISA, SRID and purity corrected BCA results from the study are also tabulated in Table 2.

**Table 2:** Tabulated Results from Blinded Comparison.

Protein	Type	Capture mAb	ToC (µg/mL)	Precision (% RSD)	N	ToC % SRID	SRID (µg/mL)	ELISA (µg/mL)	paBCA (µg/mL)
	CE	I	39±6	16	11	126	31	34	38
B/Brisbane	C1 Eluate	I	227±47	20	12	171	133	224	109
	BDS	I	713±77	11	12	164	434	823	672
	CE	G	0.42±0.2	48	7	2	18	<2.5	37
B/Mass	C1 Eluate	G	17±7	39	4	21	80	8	212
	C2 Eluate	G	512±139	27	7	95	537	191	694
	BDS	G	367±81	22	5	130	282	566	569
	CE	A	28±16	56	10	NA	NA	49	104
A/H1 CA	C1 Eluate	A	261±39	15	5	121	216	122	189
	C2 Eluate	A	3300±440^a^	13	6	516	640	2479	1561
	BDS	A	793±91	12	5	235	337	599	504
A/H3 TX Wild Type	CE	D	11±5	40	8	12	94	22	46
	C1 Eluate	D	170±15	9.0	6	49	344	138	273
	C2 Eluate	D	776±83	11	6	68	1139	716	754
	BDS	D	172±10	5.9	6	34	501	534	538
A/H3 TX Modified	CE	D	35±8	22	7	30	118	19	50
	C1 Eluate	D	254±50	19	8	33	775	113	372
	C2 Eluate	D	1115±168	15	6	44	2531	291	1003
	BDS	D	506±36	7.1	2	75	674	339	499

CE represents crude extract, C1 is column 1, C2 is column 2, and BDS represents Bulk Drug Substance.

a Value identified as a statistical outlier and not included in the ToC comparison with SRID and BCA.

### Comparison of Flu-ToC and SRID

**Figure 4:**
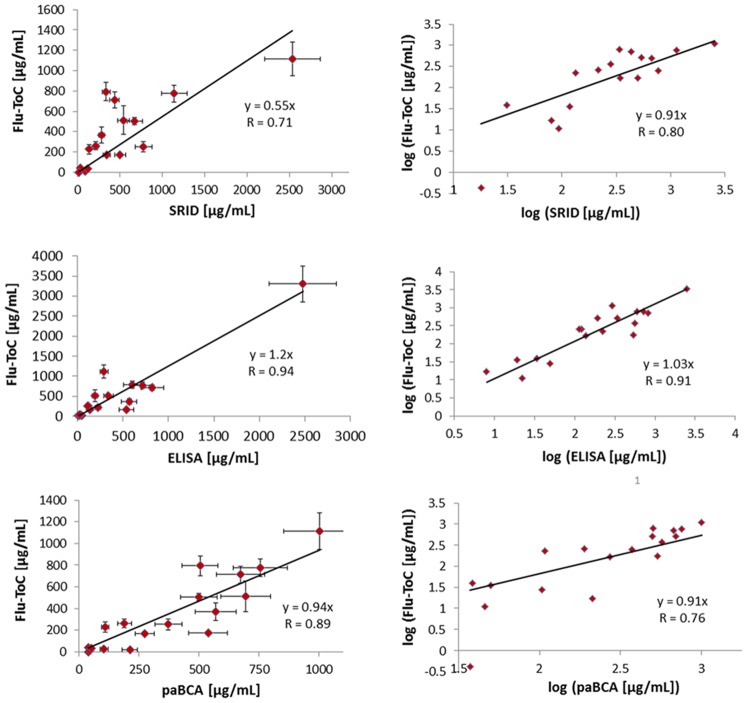
The top panel shows Flu-TOC results with measured error (±σ) plotted against SRID results. The relative error for SRID measurements is fixed at 13% based on experience (PSC). The right graph is log(Flu-ToC) vs. log(SRID). In both graphs, the solid line represents a linear regression to the data with a 0,0 intercept. The slopes and correlation coefficients are specified on the respective graphs. One data point (highlighted in yellow in Table 2) was identified as a statistical outlier and was excluded from the analysis. Middle panel shows similar comparisons to ELISA with 15% relative error. Bottom panel summarizes ToC comparison to paBCA with 15% relative error.

Since SRID is accepted as a measurement of vaccine potency, it is essential that an alternative potency method correlates with SRID to determine the potency of final vaccine formulations. **Figure 4** (top panel) summarizes the comparison of Flu-ToC to SRID in linear space (left graph) and log space (right graph). Measured error is included for Flu-ToC and 13% relative error is assumed for SRID (based on measurement experience at PSC). The solid lines in the figure represent linear regressions to the data fit through 0,0 in order to enable more accurate assessment of residuals. One data point was excluded as an identified statistical outlier based on a standard residuals analysis (standardized residual >>2). In addition, both Flu-ToC and ELISA yielded much higher values for the A/CA column 2 eluate compared to the SRID value; therefore, we also excluded that sample from the analysis. The linear regression (left panel) exhibits a slope of 0.55 and a Pearson’s correlation coefficient (R) of 0.71. The statistical significance of the correlation was assessed using the t-test for regression. In this case, t(15) = 3.9, p<0.002, which is sufficient to reject the null hypothesis, indicating a direct correlation. A slope of 0.55 indicates that Flu-ToC yield a lower measured HA content that SRID. One common method to quantify the difference between the measured values is to evaluate “percent of SRID” (i.e., (Flu-ToC value/SRID value)*100), which is summarized in column 6 of Table 2. On average, the Flu-ToC value is ∼83% of the SRID value (excluding the statistical outlier highlighted in yellow within the table). Another way to assess the difference is the error factor histogram, which shows that approximately half of the Flu-ToC values are within a factor of 2 of the SRID value. While Flu-ToC measures on average a slightly lower HA content than SRID, the observation of a reliable, linear correlation is of highest importance as a correction factor can be applied if needed.

To determine whether or not the relationship between Flu-ToC and SRID is indeed linear, we evaluated the slope of a log-log plot, which should equal 1 for a truly linear relationship. As shown in the upper right panel of Figure 4, the slope determined from regression to the log-log plot is 0.91, which is supportive of a linear relationship. Taken together, the observed trends lead us to conclude that there is a linear correlation between the rHA antigen potency as measured by Flu-ToC and by SRID.

### Comparison of Flu-ToC and ELISA

The middle panel of Figure 4 shows plots of HA content as determined Flu-ToC versus ELISA in linear space (left graph) and log space (right graph). The measurement error for Flu-ToC is included and ±15% relative error is assumed for ELISA (based on measurement experience at PSC). Eighteen of the 19 samples tested are included in the plots. One sample was not included as the concentration measured by Flu-ToC was below the quantification limit for ELISA. Based on the residuals analysis, no data point could be discarded as an outlier. The linear regression analysis of the Flu-ToC vs. ELISA plot (left panel) yields a slope of 1.2 and an R value of 0.94, indicating a high degree of correlation. An analysis of the percent of ELISA (i.e., (Flu-ToC value/ELISA value)*100) yields an average of 147%; meaning that the Flu-ToC measured HA content is ∼50 higher than the ELISA measured HA content in this study. However, as shown in the right graph of Figure 4 (middle panel), a log(Flu-ToC) versus log(ELISA) plot yields a slope of 1 with an R value of 0.91, indicating a strong linear correlation between rHA potency as measured by Flu-ToC and ELISA.

### Comparison of Flu-ToC and paBCA

The Bicinchoninic Acid assay is widely used in the flu vaccine industry as a non-specific measure of total protein content. When combined with SDS-PAGE densitometry, which is used to determine purity of the protein of interest, BCA can serve as a means to create a primary standard reference antigen; CBER employs a similar approach to characterize seasonal reference antigens. It is therefore important that alternatives to SRID exhibit good correlation with HA content measured by purity-adjusted BCA (paBCA). The right-most column in Table 2 summarizes the paBCA values for this study. The results from linear regression analysis of Flu-ToC and SRID plotted against the paBCA values are shown in the bottom panel of Figure 4. The statistical outlier identified in the correlation of Flu-ToC and SRID (i.e., SRID 640 µg/mL, Flu-ToC 3,300 µg/mL) was not included in the regression. The plot of Flu-ToC versus paBCA has a slope of 0.94 and an R value of 0.89, indicating good correlation and nearly equivalent measured HA content. The average percent of paBCA (i.e., (Flu-ToC value/paBCA value)*100) is 81%. The slope from a log-log plot regression is 0.91, with an R value of 0.76, indicating an essentially linear relationship.

**Table 3** is a summary of the correlation analysis and calculated ratios for Flu-ToC relative to SRID, ELISA, and paBCA. Since the HA content measured by Flu-ToC and SRID, ELISA, and paBCA is generally within a factor of 2 and there is reasonable correlation of results between Flu-ToC and those assays, we contend that Flu-ToC has potential as a reliable HA potency assay.

**Table 3:** Tabulated Results from Correlation Analysis.

	Linear Regression Results		Log-Log Regression Results		Ratio*
ToC vs:	Slope	R	Slope	R	(ToC/Other)
**SRID**	0.55	0.71	0.91	0.80	83±66%
**ELISA**	1.2	0.94	1.03	0.91	147±88%
**paBCA**	0.94	0.89	0.91	0.76	81±54%

### Multiplex Analysis

**Figure 5:**
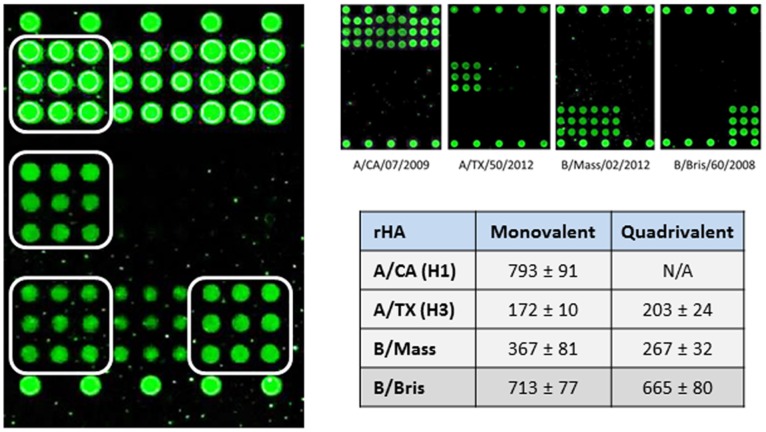
The left panel shows a representative fluorescence image of multiplexed quantification of all components within a quadrivalent formulation. The white boxes highlight the mAbs used for quantification of each strain. Right panel (top) shows representative fluorescence images for individual BDS samples. Right panel (bottom) is a comparison of results obtained from quantification of monovalent BDS and each component in a quadrivalent formulation constructed from individual BDS. The multiplex study was conducted 2 months after the monovalent study. For this particular data set, the concentration of a new H1 label was too high and the rHA H1 could not be quantified. Error for monovalent is as measured from replicate studies. Error for quadrivalent is 12% RSD, based on the precision determined for multiple measurements of BDS samples (see text for details). Within error the two sets of results are equivalent.

A significant advantage of Flu-ToC is the ability to conduct simultaneous analysis of all components in the drug product. **Figure 5** is a representative image from multiplex analysis of all components within a quadrivalent formulation (constructed from a mixture of monovalent BDS). The white boxes in the figure outline the mAbs used to quantify each component. Multiplex quantification was conducted 2 months after the initial measurements but, with the exception of A/CA HA for which a new label antibody was used for detection, within error the results were equivalent to those reported for the monovalent BDS (see Table in Figure 5). In addition, being able to employ Flu-ToC at all stages of vaccine production, from the crudest samples through the finished drug product, is of considerable benefit in regulated environments.

### Robust Response to Older Strains

Another major goal for the Flu-ToC platform is to reduce and eventually eliminate the need for development of new antisera or antibodies each time there is a strain change. In the development of Flu-ToC, the capture and label antibodies were carefully selected from commercially available materials based on their broad reactivity for the respective subtypes to specific subtypes. As noted by Bodle et al. [19], many subtype specific mAbs exhibit reliable binding for a number of seasons. In fact, they determined that despite antigenic drift resulting in 22 strain changes during the time period from 2000 to 2011, only 11 changes to select mAbs would have been required [19]. While it is not possible to predict whether or not these the broadly reactive mAbs included in v1.0 of the Flu-ToC assay will respond to future strains, we can judge their robustness in a retrospective analysis.

**Figure 6:**
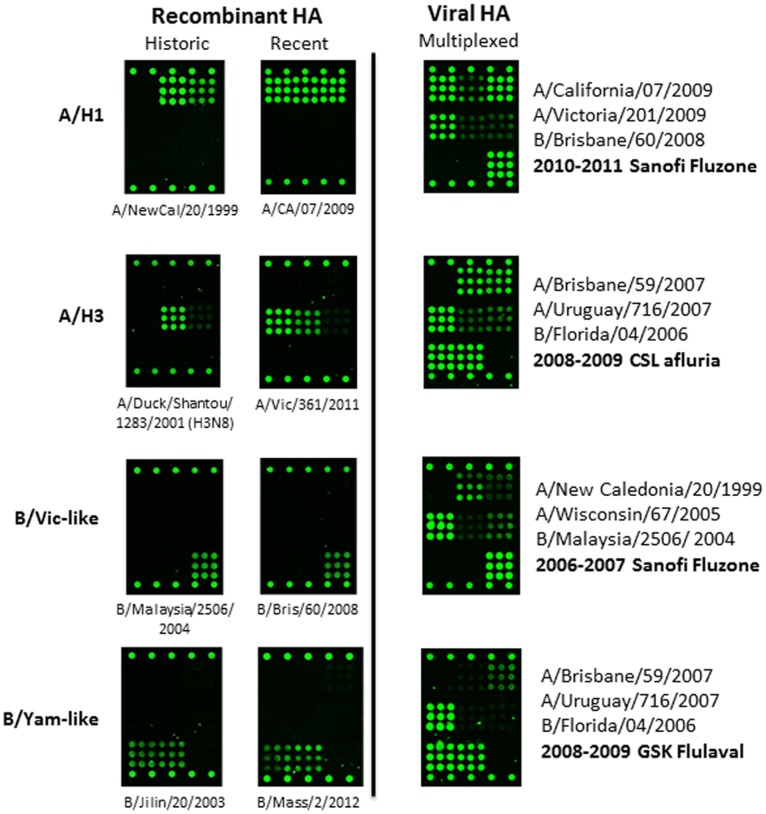
Representative fluorescence images of rHA and split virus vaccines (right column) from strains ranging over a broad time period. The originating strain designations are listed below the respective images for rHA and to the right side for the viral vaccines.

To test the response to antigenic drift, older strains of both purified recombinant proteins (Protein Sciences) as well as archived vaccines (BEI Resources) derived from split viruses were analyzed by Flu-Toc. Representative fluorescence images are displayed in **Figure 6** **.** Recombinant proteins representing a 10 year time period were readily detected on v1.0 of the array, as were all components of split virus vaccines from the time period covering 2008–2012 (not all data shown). Quantification would have been possible with appropriate reference antigens in hand. As an example, the A/CA/07/2009 HA content was determined for 7 trivalent vaccines produced during the time period of 2009–2011 since we were able to use a currently available CBER reference antigen (X-181, Lot H1-Ag-1107, CBER). In this case, calibration required serial dilution of the reference antigen lysed with Zwittergent 3–14. Flu-ToC analysis was conducted with Zwittergent 3–14 present at 1% in all samples. An average HA content of 33±6 µg/mL was measured, which corresponds to ∼112% of the released SRID value. While the Flu-ToC value is a bit high, it is certainly reasonable. Overall, this retrospective analysis indicates that the antibody panel used for v1.0 is robust and it may be useful for quantification of HA for multiple years.

## Conclusions

A blinded comparison of Flu-ToC to other influenza HA quantification methods demonstrated that this new technique exhibits reasonable correlation with purity-adjusted BCA, ELISA, and SRID for both purified and crude samples. As an alternative to these more established methods, the results show that Flu-ToC offers distinct advantages, including: i) eliminating interlaboratory variations due to in-house preparation of gels and plates, ii) eliminating the reliance on seasonal reference antisera through the use of a panel of broadly reactive but subtype specific mAbs, iii) improved reproducibility and precision attributed to the use of purified monoclonal antibodies (rather than antisera) and signal normalization based on an internal control, and iv) the ability to quantify both mono- and multi-valent vaccine formulations on the same multiplexed platform with significant reagent and time savings.
